# British Nurses and Nursing. A Campaign of Misrepresentation and Personal Ambitions

**Published:** 1917-07-07

**Authors:** 


### THE EDITOR'S LETTER-BOX.

### British Nurses and Nursing.

### A Campaign of Misrepresentation and Personal Ambitions.

*To the Editor of* THE HOSPITAL.

SIR,—A correspondence in the *Sunday Times* and the letters of those who represent the Central Committee of Divide the Nursing Profession or Perish, are self-revealing and destructive.

A lady of means, who is not a fully trained nurse, Miss Eden, publishes a letter in the issue of June 24 which is directed against the Nation's Fund for Nurses as *charity*. This is sheer nonsense, seeing it is on all-fours with the plan pursued for the endowment of many of the great Colleges at our Universities, for the creation of Exhibitions and Building Funds for our public schools, and Many Other Public Enterprises for the Uplifting of People of All Classes. The late Bishop of Winchester demonstrated in an eloquent speech forty years ago that if eleemosynary gifts of this kind were to be regarded as charity, then there was probably not a man or woman in the nation from Queen Victoria downwards, who held any position or had attained a reputation, who did not owe much of his success in life to the knowledge, training and opportunities given him through eleemosynary gifts, which were not charity, but honourable national endowments, fruitful in benefit to the nation and its highest progress. Both the Medical and Legal Professions have Great Funds derived from Eleemosynary Gifts.

Other letters are a covert attempt to further the policy of setting the nurses against their matrons and their training-schools by falsely maintaining that the only possible representation of the nurses on a democratic principle is for them to give their representation, *i.e.* their votes, to a number of Small Societies which the Dividers are trying to build up and unite in the Central Committee. In the proper meaning of the words they have really no representative character, being relatively small societies specially created by a party consisting largely of a few friends, with the hope of thus obtaining entire control of the nursing profession. At the outset these societies included the Royal British Nurses' Association and the Association for the Promotion of the Registration of Nurses in Scotland, which two bodies of nurses had a representative character and numbered thousands of nurses, *i.e.* many more, probably, than all the remaining so-called societies put together. The Divide the Profession Party's cry is for democratic control for the nurses; but the hollow humbug of it in this connection is demonstrated by the fact, that when the Royal British Nurses' Association and the Association for the Promotion of the Registration of Nurses in Scotland protested against the illegal action of the Council of the Central Committee, in suddenly breaking with the College without first consulting the members of each of what Miss Eden denominates "the organised nurses' societies," one society of the Divide Party unanimously moved the Central Committee to eject the Royal British Nurses' Association and the Association for the Promotion of the Registration of Nurses in Scotland from the few organised nurses' societies whose elected representatives collectively constitute the Central Committee.

Miss Eden, however, has the temerity to write in this connection that she and Mrs. Bedford Fenwick are fighting for a foundation principle—the right to appoint representatives; but in practice, as I have shown, this statement is refuted by the recent action of the Society for the State Registration of Nurses—in demanding the expulsion of the R.B.N.A. and the A.P.R.N.S. from the Central Committee because they have upheld this identical foundation principle. The Central Committee may know, as Miss Eden claims, that what it asks for is right; but, assuming that she is correct, this Committee evidently does what is wrong in an illegal and autocratic manner directly it suits the purpose of those who control its policy to do so. It should be manifest to nurses, therefore, that the claims in the *Sunday Times* correspondence (*a*) as to the principle of representation; and (*b*) as to the breaking away of the Central Committee from the College and the responsibility for that step, have no justification in fact. Indeed, Miss Eden, in a letter in the *Common Cause* of June 15, advances the exact contrary to the facts and the evidence.

British nurses who value their profession and desire its speedy organisation and unity—that is, more than nine-tenths of the whole number—are tired and disgusted with the repeated publication of the absurdities here indicated, which are being circulated through the *Sunday Times* and other lay papers, to secure something other than the welfare of the trained nurse. The truth is, every member of the British Nursing Profession who has received her training in a properly constituted training-school values her connection with it, retains an affection for her teachers, and acquires the habit of going back, as University men go back, and Public School men return to their School or their College, for refreshment and pleasant memories when tired by their daily work in the battle of life.

Now, I am convinced, is the time for every British nurse to insist upon the importance of loyalty, and to uphold those through whose efforts, teaching, and work the British nursing profession has become the leading centre of the best nursing to be found in the world. By joining the College now every nurse will display character and pluck, for she so takes upon herself a full and proper measure of responsibility, and shows her confidence in those to whom she owes so much and who have proved themselves to be her friends and best helpers on many occasions in the past. By this step every British nurse at the same time promotes her own interests and adopts the best course to hasten the Parliamentary recognition of the full status of every trained British nurse and the uniting and uplifting of her profession.—Yours truly,

A LIFELONG FRIEND OF NURSES.

